# Bioaugmentation Improves Phytoprotection in *Halimione portulacoides* Exposed to Mild Salt Stress: Perspectives for Salinity Tolerance Improvement

**DOI:** 10.3390/plants11081055

**Published:** 2022-04-13

**Authors:** João Carreiras, Isabel Caçador, Bernardo Duarte

**Affiliations:** 1 MARE—Marine and Environmental Sciences Centre, ARNET–Aquatic Research Infrastructure Network Associated Laboratory, Faculdade de Ciências, Universidade de Lisboa, Campo Grande, 1749-016 Lisbon, Portugal; jgcarreiras@fc.ul.pt (J.C.); micacador@fc.ul.pt (I.C.); 2 Departamento de Biologia Vegetal, Faculdade de Ciências, Universidade de Lisboa, Campo Grande, 1749-016 Lisboa, Portugal

**Keywords:** halophyte, rhizobacteria, PGPR, root inoculation, osmotic stress

## Abstract

Plant growth-promoting rhizobacteria (PGPR) can promote plant growth through mechanisms such as mineral phosphates solubilization, biological N_2_ fixation and siderophores and phytohormones production. The present work aims to evaluate the physiological fitness improvement by PGPR in *Halimione portulacoides* under mild and severe salt stress. PGPR-inoculated plants showed improved energy use efficiencies, namely in terms of the trapped and electron transport energy fluxes, and reduced energy dissipation. Allied to this, under mild stress, inoculated plants exhibited a significant reduction of the Na and Cl root concentrations, accompanied by a significant increase in K and Ca leaf content. This ion profile reshaping was intrinsically connected with an increased leaf proline content in inoculated plants. Moreover, bioaugmented plants showed an increased photoprotection ability, through lutein and zeaxanthin leaf concentration increase, allowing plants to cope with potentially photoinhibition conditions. Reduced Na leaf uptake in inoculated plants, apparently reduced the oxidative stress degree as observed by the superoxide dismutase and peroxidase activity reduction. Additionally, a reduced lipid peroxidation degree was observed in inoculated plants, while compared to their non-inoculated counterparts. These results, point out an important role of bioaugmentation in promoting plant fitness and improving salt tolerance, with a great potential for applications in biosaline agriculture and salinized soil restoration.

## 1. Introduction

It is understood that a rapid climate change is taking place worldwide with alarming environmental and economic implications, notably, sea-level and temperature rise, an increase in the frequency and intensity of extreme climate events, such as droughts, floods, and storms, resulting in water and land salinity variation [1]. Simultaneously, it is predicted that by 2050 the world population will reach 9.7 billion, and consequently, global food demand is expected to increase by 35% to 56%, and freshwater demands for irrigation are predicted to increase by 19% [2]. Moreover, an estimated 33% of the world’s irrigated agricultural lands are suffering from high soil salinity a reduction of 1 to 2% of the agricultural area every year [3,4]. Soil salinization reduces crop yields and can affect the performance of other ecosystems services. This often-neglected continuous degradation is exacerbated by inadequate agricultural and water management practices [5]. These aspects impose severe salinity-induced constraints on crop yield worldwide, thus causing major socioeconomic costs and a probable impact on future global food security [6].

Current research has to aim at identifying and testing potential solutions to meet future agricultural demands. Usually, salt stress is significantly limiting productivity of plants, upsetting every major crop development and productivity. Most of the world’s cash crop plants, when exposed to a NaCl concentration from 40 mM to 200 mM, become severely damaged, and most do not survive [7]. On the other hand, and in contrast, halophytes can not only survive but also thrive and be highly productive under these same saline conditions. Halophytes, which constitute about 1% of the Earth’s flora, are defined as plant species that can grow and reproduce under salinity concentrations of over 200 mM NaCl [8]. These plants are known to have an important role in the stability and protection of coastal and wetland habitats, maintaining ecological stability, and providing various and unique globally relevant ecological and economic services. They also have enormous potential to aid soil restoration, water purification, and agricultural development [9]. Phytoremediation in saline-sodic soils was found to be an inexpensive solution to regulate environments and is found to be a potential substitute for chemical amelioration [10]. In line with this approach, several halophyte species can remove, transfer, or stabilize salt, metals, pharmaceutical products, pesticides, cyanotoxins, and nanoparticles from the soil and groundwater in an efficient, low-cost, easy-to-use, and eco-friendly way [11]. Furthermore, increasing demand for irrigation along with a growing demand for improved agricultural practices makes it obvious that there is a need for sustainable crop production. The use of seawater irrigation for biomass production is a strategy to cope with the loss of arable land and available water resources. However, this proposes the growth of halophytes as a sustainable edible crop (cash crops) [12]. *Halimione portulacoides* (L.) Aellen is a halophyte that is a good candidate for such an approach. It is one of the most widely spread and abundant species found in the Mediterranean marshes, a C3 succulent that belongs to the Amaranthaceae family [13,14]. Moreover, this species was previously used in biosaline agriculture and salinized soil reclamation, displaying an added-value nutritional profile [15], which reinforces its potential application and the usefulness of a potential improvement of its tolerance throughout bioaugmentation approaches.

Plant-growth promoting rhizobacteria (PGPR) are beneficial soil bacteria that by mutualistic interactions directly and/or indirectly improves plant growth and development [16]. It has been shown that PGPR, as well as microbial endophytes, facilitate stress resistance and tolerance mechanisms by a wide spectrum of mechanisms [17,18]. These may include fixation of atmospheric nitrogen, phosphate solubilization, iron acquisition, balancing the levels of plant hormones, and prevention of pathogen attack [19,20,21,22]. In agricultural practice, the application of PGPR consortia, instead of a single strain, is increasingly more common to achieve or elicit all the desired responses [23,24]. Additionally, several variables, such as temperature, water availability, soil characteristics, plant species, and so on, limit the effectiveness of PGPR use [25]. For example, it was found that while an increase in soil salinity could not affect rhizobacteria growth, it could induce a loss in the production of their plant-growth promoting traits [26]. Nonetheless, several studies have not only identified salt tolerate PGPR but also associated PGPR consortia specific for salt-stressed plants [18,24]. Halophytes like *H. portulacoides* can inhabit high salinity areas, but it is well-known that even salt-tolerant species have their NaCl limitations. Exceeding the tolerance limit provokes physiological and biochemical alterations, affecting growth and potentially causing death [8]. Despite this, most studies on PGPR-inducing salt-stress resistance have been undertaken in salt-sensitive plants, while information regarding PGPR application to improve salinity tolerance in halophytes is still scarce.

For this study, PGPRs species most abundant in coastal salt marshes, specifically *Bacillus aryabhattai*, *Stenotrophomonas rhizophila*, *Pseudomonas oryzihabitans*, and *Salinicola endophyticus* have been selected in order to build a potentially effective consortium. Our choice was supported by recent promising findings on salt stress amelioration in halophyte plants [24,27,28,29]. The consortium was assembled using high salinity resistant rhizobacteria with different plant-growth promotion activities and traits that complement and reinforce each other as seen in Table 1. This work aims at evaluating the physiological fitness and PGPR-mediated improvement of stress tolerance of *H. portulacoides* under mild and severe salt stress. Therefore we have inoculated experimental plants with a PGPB consortium composed of rhizobacteria with different plant-growth promoting traits and have exposed them to mild and severe salinity levels. We have analysed the photochemical performance and biochemical traits. We will discuss the potential PGPR have to ameliorate adverse effects of salinity on *H. portulacoides*. The question is at what degree the biotechnological potential for re-vegetation of salt-affected soils can be improved or actually be completely restored.

## 2. Results

### 2.1. Photochemical Processes

**Figure 1:**
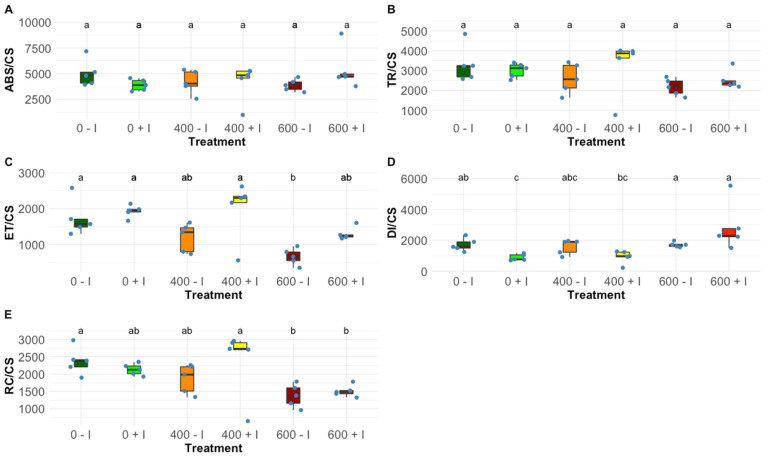
Phenomological energetic parameters, (**A**) absorbed energy flux (ABS/CS), (**B**) trapped energy flux (TR/CS), (**C**) electron transport energy flux (ET/CS), (**D**) dissipation energy flux (DI/CS) and (**E**) oxidized reaction centres (RC/CS) on a cross-section basis, in non-inoculated and inoculated *Halimione portulacoides* dark-adapted leaves (N = 5), along with the tested NaCl concentrations. Letters indicate significant differences between treatments at *p* < 0.05.

When exposed to a salinity gradient, *H. portulacoides* plants displayed significant differences in key energy transduction flux per leaf cross-section due to rhizobacterial inoculation. No significant differences were found in terms of absorbed energy flux (ABS/CS; Figure 1A) and trapped energy flux (TR/CS; Figure 1B), neither between inoculated and non-inoculated plants nor between salinity levels exposure. Salinity reduced significantly the electron transport energy flux (ET/CS; Figure 1C), especially at 600 mM. However, non-significantly it is possible to observe a tendency for an increased ET/CS energy flux in inoculated plants. The dissipation energy flux (DI/CS; Figure 1D) was not affected significantly by the inoculation status of the plants, apart from the plants cultivated under 0 mM NaCl. The higher salinity levels tested led to a decreasing tendency in the number of oxidized reaction centres (RC/CS; Figure 1E).

**Figure 2:**
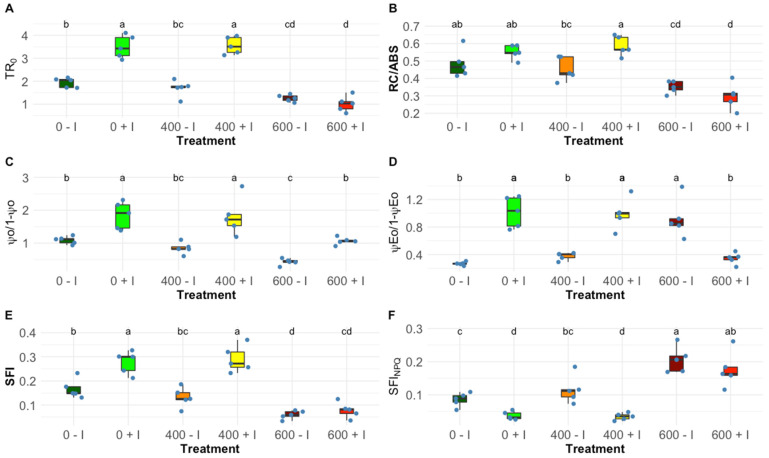
OJIP-derived parameters, (**A**) contribution or partial performance due to the light reactions for primary photochemistry (TR_0_/DI_0_), (**B**) reaction centre II density within the antenna chlorophyll bed of PS II (RC/ABS), (**C**) the contribution of the dark reactions from quinone A to plastoquinone (ψ_0_/(1 − ψ_0_)), (**D**) the equilibrium constant for the redox reactions between PS II and PS I (ψ_E0_/(1 − ψ_E0_)), (**E**) structural and functional index for photosynthesis (SFI) and (**F**) non-photosynthetic or dissipation structural and functional index (SFI_NPQ_) in non-inoculated and inoculated *Halimione portulacoides* dark-adapted leaves (N = 5), along with the tested NaCl concentrations. Letters indicate significant differences between treatments at *p* < 0.05.

Observing the OJIP-derived parameters, significant changes were found in PS II and PS I photochemical traits. Inoculated plants, at 0 and 400 mM NaCl, displayed significantly higher values in the contribution or partial performance of the light reactions for PS I (TR_0_/DI_0_; Figure 2A) and the contribution of dark reactions from Q_A_^−^ to plastoquinone (ψ_0_/(1 − ψ_0_); Figure 2C), this variation was also found in the PS II/PS I redox equilibrium constant (ψ_E0_/(1 − ψ_E0_); Figure 2D), favouring PS II. A similar trend was found in the reaction centre density within the PS II antenna chlorophyll bed but was significant only at 400 mM NaCl (RC/ABS; Figure 2B). Regarding the two integrative indexes, significant and distinct values were visible, when treated with 0 mM and 400 mM salt concentrations, the structural and functional index (SFI; Figure 2E) was significantly higher in the inoculated plants and, as expected, lower in the non-photochemical or dissipation structural and functional index (SFI_NPQ_; Figure 2F). At 600 mM NaCl, these variables did not show a significant variation between non-inoculated and inoculated samples.

### 2.2. Na, K, Ca and Cl Accumulation in Leaf, Stem, and Root Tissues

**Figure 3:**
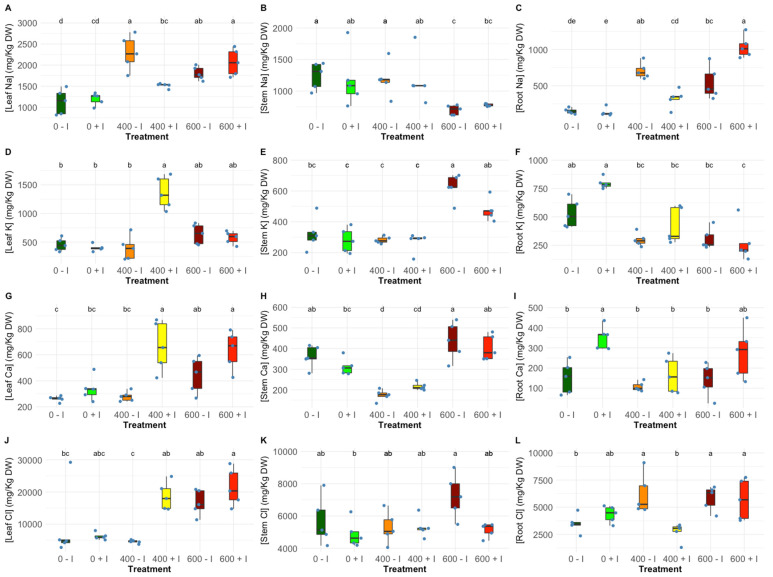
(**A**–**C**) Sodium (Na), (**D**–**F**) potassium (K), (**G**–**I**) calcium (Ca) and (**J**–**L**) chloride (Cl) concentration (mg/Kg) in non-inoculated and inoculated *Halimione portulacoides* (N = 5) leaf, stem and root tissues (N = 5), along with the tested NaCl concentrations. Letters indicate significant differences between treatments at *p* < 0.05.

Elemental concentration inside the plant tissues exhibited differential allocation upon the application of the tested salt regimes, displaying some expected significant variation amongst NaCl concentrations and between PGPR-treated plants. Nonetheless, in the stem tissues, there were no significant differences found between non-inoculated and inoculated *H. portulacoides*, leaving the intra-salt treatment variation to be found only in leaf and root tissues (Figure 3B,E,H,K). Ion accumulation in this halophyte appears to have a similar tendency within salt treatments except at 400 mM NaCl, where inoculated plants, when compared to non-inoculated plants, show a significantly lower quantity of Na and a significantly higher Cl in the leaf tissues (Figure 3A,G), and displayed significant lower values of Na and Cl in the root tissues (Figure 3C,L). Additionally, at 400 mM NaCl, it was observed that there was a significant increase in the K and Ca in the leaves of the inoculated plants (Figure 3D,G), whereas root Ca was found to be significantly higher in inoculated plants at 0 mM NaCl (Figure 3I); nonetheless, the root K accumulation was not affected by inoculation treatment (Figure 3F).

### 2.3. Photosynthetic Pigments Profile

**Figure 4:**
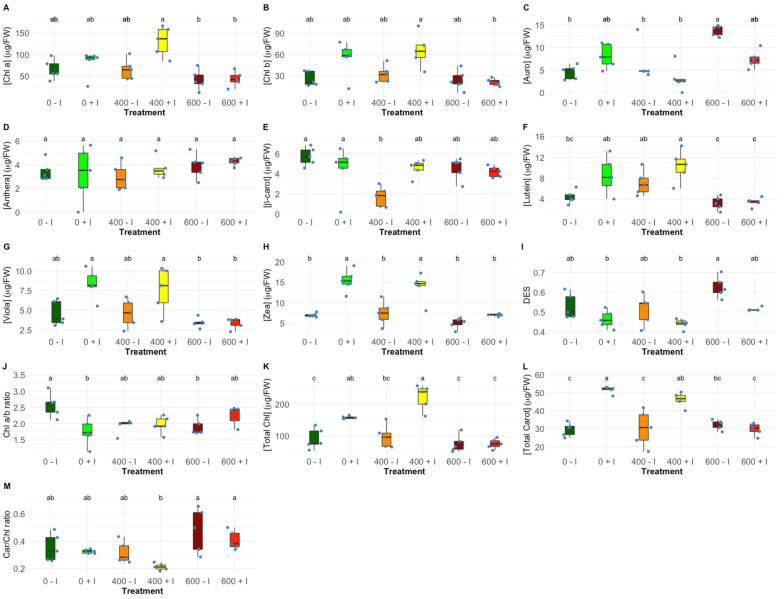
Leaves pigment relative concentrations (µg g^−1^ FW), (**A**) chlorophyll *a* (Chl a), (**B**) chlorophyll *b* (Chl b), (**C**) auroxanthin (Auro), (**D**) antheraxanthin (Anthera), (**E**) β-carotenoids (β-carot), (**F**) lutein, (**G**) violaxanthin (Viola) and (**H**) zeaxanthin (Zea), (**I**) de-epoxidation state (DES), (**J**) chlorophyll *a*/*b* ratio (Chl *a/b* ratio), total (**K**) chlorophyll and (**L**) carotenoid (µg g^−1^ FW), and (**M**) total carotenoid to total chlorophyll ratio (Car/Chl ratio) in non-inoculated and inoculated *Halimione portulacoides* individuals (N = 5), along with the tested NaCl concentrations. Letters indicate significant differences between treatments at *p* < 0.05.

Salt treatments of non-inoculated, as well as inoculated *H. portulacoides* plants, resulted in significant changes of both individual pigment concentrations and concentration ratios between pigments. No significative differences were recorded in chlorophyll *a* and *b* contents between inoculated and non-inoculated samples at all tested NaCl concentrations (Figure 4A,B, Chl *a*, Chl *b*, respectively), while significative differences were recorded in total chlorophyll and carotenoids, at 0 and 400 mM NaCl (Figure 4K,L). When analysing the auroxanthin concentration in leaves, no significant differences between salt treatments were found (Figure 4C). Additionally, zeaxanthin concentration was found to be significantly higher in the bioaugmented plants when subjected to 0 and 400 mM NaCl (Figure 4H). Similar, but not statistically significant, variations were detected in the concentration of lutein (Figure 4F) and violaxanthin (Figure 4G). Non-inoculated at 400 mM NaCl showed a significant decrease in β-carotene concentration (Figure 4E). Antheraxanthin did not exhibit observable differences between inoculate treated plants (Figure 4D). Regarding the pigment ratios in the leaves exposed to the different salinities, bacterial inoculation led to a lower chlorophyll *a*/*b* ratio (Chl *a/b* ratio) at 0 mM NaCl (Figure 4J). On the other hand, no significant differences in the total carotenoid to total chlorophyll ratio (Figure 4M, Car/Chl ratio) and de-epoxidation state (Figure 4I, DES) were noted between non-inoculated and inoculated *H. portulacoides* along with salt treatments.

### 2.4. Antioxidant Enzymatic Activities

**Figure 5:**
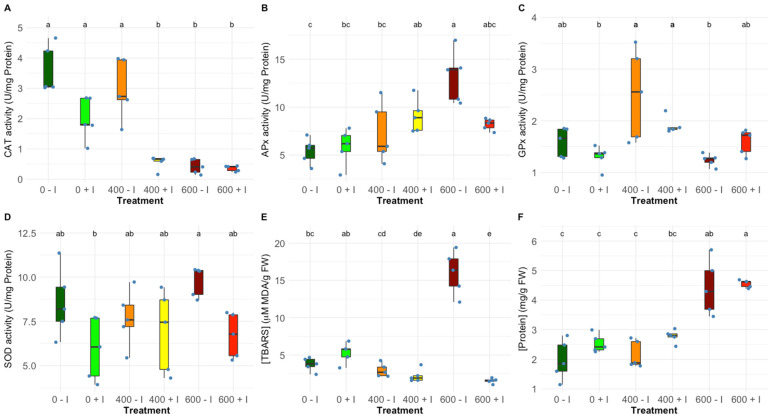
Oxidative stress biomarkers, (**A**) catalase (CAT), (**B**) ascorbate peroxidase (APx), (**C**) guaiacol peroxidase (GPx), and (**D**) superoxide dismutase (SOD) activities (U mg^−1^ protein) and (**E**) thiobarbituric acid reactive substances (TBARS; µM MDA g^−1^ FW) and (**F**) total protein content (Protein; µg Protein g^−1^FW) in non-inoculated and inoculated *Halimione portulacoides* individuals (N = 5), along with the tested NaCl concentrations. Letters indicate significant differences between treatments at *p* < 0.05.

Regarding the antioxidant enzyme activity in the leaf, PGPB inoculation led to a dissimilar response to salinity changes. Catalase activity (CAT) presented a highly significant decrease when salt concentrations increase; nevertheless, at 400 mM NaCl, the non-inoculated plants did not display a significant reduction (Figure 5A). Contrarily ascorbate peroxidase activity (APx) exhibited an increasing trend through salt treatments without significant differences between inoculated and non-inoculated plants (Figure 5B). Guaiacol peroxidase activity (GPx) and superoxide dismutase activity (SOD) did not show any significant variations between the non-inoculated and the inoculated groups (Figure 5C,D). Analysing the concentration of thiobarbituric acid reactive substances (TBARS), non-inoculated plants exhibited a significant increase when exposed to 600 mM NaCl (Figure 5E). Finally, regarding the total protein content of the leaves, an increasing tendency across the increasing salinities was observed in both sample groups and no significance was found in intra-salinity treatments.

### 2.5. Proline Quantification

**Figure 6:**
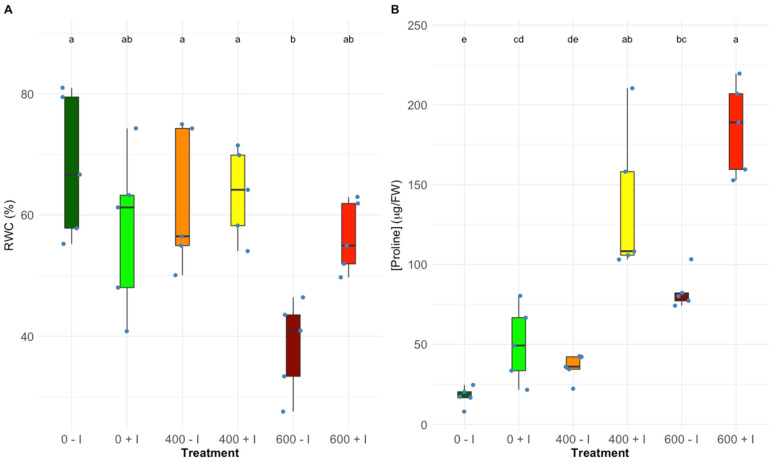
Leaves, (**A**) relative water content (RWC) and proline concentration (**B**) of non-inoculated and inoculated *Halimione portulacoides* individuals (average ± standard error, N = 5), along with the tested NaCl concentrations. Letters indicate significant differences between treatments at *p* < 0.05.

Leaf water content was very similar in all tested treatments except for the non-inoculated plants exposed to the highest salinity level, which showed comparatively low leaf water content when compared to the remaining treatments (Figure 6A). It is worth noticing that under the highest salinity level, inoculated plants maintained their leaf water content. Proline content in both *H. portulacoides* groups exhibited an upward trend following the NaCl raise (Figure 6B). However, when comparing non-inoculated plants to PGPR-inoculated plants, a significantly higher proline concentration was induced by every salt treatment (0, 400, and 600 mM NaCl).

## 3. Discussion

The Intergovernmental Panel on Climate Change (IPCC) report predicts a worldwide environmental change with alarming social, economic, and environmental consequences [1]. This will be particularly felt in coastal regions. Here, adverse effects of the temperature rise, expanding drought seasons, intensification of storm surges, and sea-level rises will be aggravated by intensive agricultural practices of the XXI century. Therefore, it can be presumed that water and soil salinization is one of the major worldwide plant stressors, and increasingly so [30]. Plant growth-promoting bacteria (PGPR) have been widely recognized to be a great tool for the augmentation of crop productivity and the improvement of the plant’s stress coping ability [31,32]. On the other hand, very little attention has been given to the enhancement of the innate tolerances found in the halophytes. The augmentation of the tolerance to excess salt is of great value considering the potential halotolerant *H. portulacoides* have as a sea-water cash crop and in the bio-reclamation of salt-affected soils [33]. In this work, bioaugmentation was achieved by root inoculation using a consortium formed by competent salt marsh PGB-bacteria that were obtained and evaluated in a previous study [24].

The traits evaluated through the chlorophyll fluorescence measurements confirmed that *H. portulacoides* is highly salt-tolerant, as expected from the findings in previous studies [34,35]. However, at the highest tested concentration (600 mM NaCl) already some signs of stress could be found. Nevertheless, the most noticeable differences were detected when comparing the non-inoculated and inoculated plants’ responses to the NaCl treatments. The photochemical analysis showed that the inoculated plants exhibited a substantial improvement in their photosystem II efficiency at mild salt stress. Inoculated *H. portulacoides*, at 400 mM NaCl, displayed higher trapped (TR/CS) and transport energy flux (ET/CS) per cross-section, the main flux responsible for chemical energy production, and available reaction centres (RC/CS) per cross-section. In addition, bioaugmentation significantly increases the reaction centre II density within the PS II antenna. This could explain the greater energy translated at the electron transport chain, from quinone A to plastoquinone, suggesting a PGPR-induced improvement in the photon capture capability as well as a more efficient light-harvesting mechanism [36]. Plastoquinone functions are the primary electron acceptor of PS II [37]. Consequently, augmentation of plastoquinone activity plays a role in the significantly improved performance of the light reactions in primary photochemistry. This results in the observed enhancement of the overall equilibrium constant for the redox reactions between PS II and PS I [38,39,40,41]. These features are reflected in the significant increase shown by the structural and functional index for photosynthesis (SFI) in the inoculated plants at 0 mM and 400 mM NaCl, and, as expected, a significantly lower non-photosynthetic or dissipation structure functional index (SFI_NPQ_) [42]. Additionally, the typical higher dissipation energy flux was observed in stressed individuals [43], and was also ameliorated in the plants inoculated with PGPB. These results revealed the PGPR consortium’s significant enhancement of the photochemical performance of the plants at 0 mM NaCl but with a special impact at 400 mM NaCl.

These differences found at the photosynthetic level can be explained, in part, by the influence exercised by the PGPR in the ionic uptake and osmotic regulation mechanisms. As expected, the increase of NaCl in the environment led to an increase in the content of Na in the *H. portulacoides* leaves and roots. This is normally associated with salt stress biomarkers due to the direct correlation between the accumulation of Na in the cell and plant salt toxicity [44,45]. Besides, the increasing Na concentration in the medium will directly compete with other essential ions like K, resulting in the impairment of cellular functions regulated by potassium, such as enzyme activation, protein production, and photosynthesis [46,47]. Nonetheless, our results, at mild salinity stress, showed that PGPR induced a significant reduction of the Na and Cl concentrations present in the roots, as well as a significant increase of the K and Ca contents in the leaf tissues. This may explain the significantly lower Na content found in the leaves of inoculated plants. This reveals superior maintenance of ionic homeostasis, indicated by the K: Na ratio in the tissues of inoculated plants. The increase of the Ca concentration in the leaves could serve as an ionic balance mechanism, allowing the cell membrane to maintain the K/Na selectivity [43]. The uptake of K and Ca due to PGPR inoculation has been demonstrated to result in the up-regulation of the antioxidant system and mitigation of salt stress-induced oxidative effects [48]. Furthermore, it has been described that rhizosphere inoculation by such halotolerant bacteria species may confer salt tolerance through sequestering Na^+^ into vacuoles, expelling Na^+^ from roots, and through the exertion of modulating attributes of the ion transporters involved [49,50].

Leaf osmotic regulation is of crucial importance in salt stress plants [51]. The increased presence of organic osmolytes is a widespread plant strategy to prevent stress-induced damage to cellular organelles [52]. In halophyte plants, to counteract soil salinity, an accumulation of L-proline in leaf tissues is commonly observed. In addition to its osmoprotectant potential [53,54], this amino acid is considered to play a pivotal role in the protection of cellular membrane structures, the activities of ROS scavenging enzymes, and the maintenance of leaf water content [55]. The proline content in leaves of *Halimione portulacoides* increased with increasing NaCl stress. This increase and concomitant leaf L-proline content were more pronounced in inoculated plants as compared to non-treated plants. The effect of the greater proline availability was clearly expressed in the leaf’s relative water content, at 600 mM NaCl. It is also known that, as a nitrogen-containing amino acid, proline expression is enhanced when nitrogen is available, this relationship may be considered an important parameter explaining the more pronounced increase of proline concentration found in the inoculated samples [56,57].

Regarding the pigment profiles, it was clear that *H. portulacoides* plants were affected by the rise in salt concentration, revealing a salinity-induced shift in the metabolic activity from photo-harvesting to photoprotection. The role of the xanthophyll cycle in the photoprotection of PSII is a well-characterized energy dissipation mechanism commonly observed in all plants [58,59]. The de-epoxidation state (DES) of luminal violaxanthin cycle pigments was found to be one of the most effective mechanisms for the dissipation of excess light energy, reducing the overload of energy within light-harvesting complexes (LHCs) [60,61,62]. Even though an increase of the de-epoxidation occurred at the highest test NaCl concentration, significant differences induced by bacterial inoculation were not found. However, the concentration of zeaxanthin was found to be significantly higher in the bio-augmented plants, at 0 mM and 400 mM NaCl. Zeaxanthin is characterized by having an important photoprotective role. It was found to enhance heat release. This parameter can be measured as non-photochemical quenching (NPQ) of excitation energy [63,64]. Lutein plays a quasi-opposite role. It has been described as functioning as a structural stabilization of antenna proteins and as a light-harvesting contributor. Due to the long half-life time of its light-activated state, it is feeding excitation energy to Chl [60,65]. At 400 mM, the higher lutein concentration found in the inoculated plants was paralleled by a significantly higher zeaxanthin concentration. These changes in the lutein and zeaxanthin concentrations allied to a significantly higher RC II density within the antenna chlorophyll bed and increased PSII antenna size, point toward an increased photoprotective ability under PGPR inoculation [39,59]. Moreover, the content of auroxanthin, a noteworthy carotenoid due to its well-described role as an effective energy quencher under salinity stress conditions in isolated LHC IIb, showed a significant increase at 600 mM NaCl in the non-inoculated *H. portulacoides* [39,66,67]. This can indicate an alleviation of the potential photoinhibition conditions due to bacterial inoculation, and thus a reduction of the photoprotective countermeasures. The chlorophyll *a*/*b* ratio displayed a significant decrease with increasing NaCl concentrations in non-inoculated plants only. This may indicate that the absorbed rate of light energy exceeds the photochemical capability [43]. Additionally, at 0 mM and 400 mM NaCl, inoculated plants experienced an amelioration effect brought about by a significant increase in the total chlorophyll concentration. This can be explained by an enhanced nitrogen fixation capability found in the PGPR inoculate. Thus, an enhanced supply of nitrogen can be assumed that will stimulate the production of photoreceptor pigments [24,68,69].

It is known that salinity-induced stress can cause the accumulation of harmful molecules in plant tissues. An excess of Na^+^ ions in the cytoplasm has a negative influence on the uptake of other ions. This can ultimately lead to the impairment of various metabolic pathways paralleled by increased production of reactive oxygen species (ROS) [68,70]. To counteract this latter effect, halophytes build up effective antioxidative mechanisms, as proficient enzyme-based systems as well as mechanisms driven by pigment conversions [71,72]. When assessing the oxidative stress biomarkers, equilibrated response tendencies to the salt gradient were found, but some noteworthy PGPR induced discrepancies were seen between the treated groups. Non-inoculated and inoculated plants showed a catalase (CAT) activity and ascorbate peroxidase (APx) activity reduction through increasing salinities; however, at 400 mM NaCl, catalase displayed significantly higher activity in the non-inoculated samples, possibly due to the also significantly higher uptake of Na^+^ in the leaves [73,74]. On the other hand, at 600 mM NaCl, superoxide dismutase (SOD) and ascorbate peroxidase activity, in non-inoculated individuals, were shown to be higher, pointing to a higher H_2_O_2_ production by SOD and subsequent metabolization by APx [75]. Nevertheless, the significant increase of lipid peroxidation products (TBARS) found in the same group suggests that in non-inoculated *H. portulacoides* when exposed to 600 mM NaCl, the peroxidasic metabolization might not be sufficient with a consequent higher ROS production and lower ROS scavenging mechanisms [74]. In summary, from the analysis of provided data, it can be proposed that root inoculation by the chosen PGPR consortium resulted in a significant amelioration effect. Phytoprotection and overall improved fitness of *H. portulacoides* were observed when exposing the plants to mild NaCl stress.

## 4. Materials and Methods

### 4.1. Sampling Sites and Plant Material Collection

Samples of *Halimione portulacoides* were collected from Tagus estuary salt marsh located on the western coast of Portugal (38°46′ N 9°05′ W). In the laboratory, plant samples were gently washed to remove dust and sediments. To make steam cuttings from the *H. portulacoides* samples, the roots and part of the stems were cut, leaving at least two nods in the stem below the lowest branch for the development of grafts. The props were hydroponically cultured in dark-walled vases filled with a nutritive solution (N:P:K4:5:7, Bo 0.01%, Cu 0.002%, Fe 0.02%, Mn 0.01%, Mo 0.001%, Zn 0.002%) and placed in a phytoclimatic chamber programmed to simulate a natural light environment using a sinusoidal function (maximum PAR 300 µmol photons m^2^ s^−1^, 16/8 h day/night rhythm, 20/18 °C day/night temperature amplitude). Plants were kept under this condition for 2 months to acclimate to the new environment and to allow root biomass growth.

### 4.2. Rhizobacteria Used for Inoculation in This Study

The rhizobacteria consortium (Table 1) was made with rhizobacteria obtained from different halophytes inhabiting the Portuguese west coast salt marshes: *B. aryabhattai* and *S. rhizophila* were collected from Horta dos Peixinhos, Aveiro (40°45′ N 8°56′ W) and *P. oryzihabitans* and *S. endophyticus* from Tagus estuary, Lisbon (38°46′ N 9°05′ W). Bulk sediments adjacent to the halophyte plants were collected by hand spade and transferred to sterile plastic bags, transported to the laboratory, and stored at 4 °C until analyses. Interstitial (pore) water was collected at the corresponding sites using Rhizon samplers (Rhizosphere Research Products, Wageningen, The Netherlands). The rhizobacteria used in the consortium formation were isolated, identified, and are described in a previous study [24].

### 4.3. Preparation of Bacterial Inoculants

To prepare the four suspensions for root inoculation, all rhizobacteria were grown separately in 250 mL Erlenmeyer flasks containing 50 mL of tryptic soy broth (TSB) medium modified by the addition of NaCl 25 gL^−1^ in a rotary shaker for 24 h (150 rpm, 30 °C). Then, cultures were centrifugated in 50 mL sterile Falcon tubes at 5000× *g*, 10 min at room temperature, and the supernatant was discarded. Pellets were washed twice with sterile physiological saline solution (NaCl 0.9% *w*/*v*) and adjusted to a final concentration of 10^8^ CFU mL^−1^ (OD600 = 1) [18]. Bacterial suspensions were then mixed, in equal amounts, to produce the final consortium suspension. To assess the potential incompatibility effects of the bacteria species used, isolates were plated together in a solid agar TSB medium, and its growth halo diameter was compared with the growth halo of each species when plated individually (data not shown). No significant differences were observed between the co-cultures and the monocultures, indicating that there was no incompatibility between the bacteria.

### 4.4. Experimental Setup and Root Inoculation

After significant root biomass development, plants were arranged into 6 groups with 5 replicates, potted individually. The first group of plants composed of a set of three groups of five plants each were inoculated with the previously prepared rhizobacterial consortium solution (Table 1), by soaking the roots, previously washed with deionized water, in the bacterial suspension for 6 h at room temperature (25 ± 1 °C). The bacterial suspension was diluted with 0.9% (*w*/*v*) saline solution to achieve a concentration of 10^7^ CFU mL^−1^, prepared as described previously [76]. The second group of plants composed of a set of three groups of five plants each were soaked similarly in 0.9% (*w*/*v*) saline solution without bacterial inoculation. This process occurred for 10 days. After this period, *H. portulacoides* individuals from both inoculated and non-inoculated groups were separated into three sets with five replicate individuals and were kept under the same described conditions. The nutrient solution was replaced and supplemented with NaCl to attain the desired target salinities (0, 400 and 600 mM NaCl):

0 mM NaCl non-Inoculated (0 − I)

0 mM NaCl Inoculated (0 + I)

400 mM NaCl non-Inoculated (400 − I)

400 mM NaCl Inoculated (400 + I)

600 mM NaCl non-Inoculated (600 − I)

600 mM NaCl Inoculated (600 + I)

Salinity exposure trials lasted for 7 days after which chlorophyll fluorescence measurements were made and consecutively and plants were harvested. Leaf samples for biochemical measurements were immediately flash-frozen in liquid N_2_ and stored at −80 °C until analysis.

### 4.5. Pulse Amplitude Modulated (PAM) Fluorometry

Modulated chlorophyll fluorescence measurements were made on attached leaves using a FluoroPen FP100 PAM (Photo System Instruments, Brno, Czech Republic). All measurements in the dark-adapted state were made after the darkening of the leaves for at least 30 min. The OJIP transient (or Kautsky curves) depicts the rate of reduction kinetics of various components of PS II. This is obtained when a dark-adapted leaf is illuminated with a saturating light intensity of 3500 µmol m^−2^ s^−1^. Then it exhibits a polyphasic rise in fluorescence (OJIP): level O represents all the open reaction centres at the onset of illumination with no reduction of Q_A_ (fluorescence intensity lasts for 10 ms); the O to J transient indicates the net photochemical reduction of Q_A_ (the stable primary electron acceptor of PS II) to Q_A_^−^ (lasts for 2 ms); the J to I transition is due to all reduced states of closed RCs such as Q_A_^−^ Q_B_^−^, Q_A_ Q_B_^2−^ and Q_A_^−^ Q_B_ H_2_ (lasts for 2–30 ms); P-step coincides with a maximum concentration of Q_A_^−^ Q_B_^2^ with plastoquinol pool maximally reduced. It also reflects a balance between the light incident at the PS II side and the rate of utilization of the chemical (potential) energy and the rate of heat dissipation [77]. Table 2 summarizes all the parameters that were calculated from the fluorometric analysis.

### 4.6. Ion Analysis

For measuring for, K, Ca, and Cl contents, aboveground and belowground organs were washed with ultra-pure water to eliminate salts from the surface of the organs. Subsequently, they were dried at 60 °C until constant weight and ground in an agate mortar. 100 mg samples of dried powdered material were subjected to acid digestion. This procedure was carried out in Teflon reactors using an acid mixture of HNO_3_:HClO_4_ (7:1, *v*/*v*) at 110 °C for 3 h. After cooling overnight, the digestion products were filtered through Whatman No. 42 (2.5 μm pore diameter) filters and diluted with distilled water to a total volume of 10 mL. Elemental quantifications were made using a TXRF instrument (S2 PICOFOX^TM^ spectrometer, Bruker Nano GmbH, Berlin, Germany). The cleaning and preparation of the TXRF quartz glass sample carriers was performed according to [78]. For analysis 5 μL of the filtered digestion or extraction product was placed in the middle of the quartz carriers. In each batch of samples, 3 quartz carriers containing mono and multi-elemental standards (Bruker Nano GmbH, Germany) were used to calibrate, assess the analytical sensitivity and detection limits, as well as quantification accuracy. Each sample was irradiated for 800 s per sample. The accuracy and precision of the analytical methodology for elemental determinations were assessed by replicate analysis of certified reference material BCR-146. Blanks and the concurrent analysis of the standard reference material were used to detect possible contamination/losses during analysis. The TXRF spectra and data evaluation was performed using the Spectra 7.8.2.0 software.

### 4.7. Pigment Profiling

Leaves from the different treatments were weighted before (FW) and after (DW) freeze-drying and this was used to calculate the leaf relative water content as RWC (%) = (FW − DW)/FW. Ground freeze-dried leaf samples were extracted with 100% acetone added and subjected to an ultra-sound bath for 1 min to ensure complete disaggregation of the leaf material. Extraction occurred in the dark for 24 h at −20 °C, after which the samples were centrifuged at 4000× *g*, 15 min at 4 °C. Absorbance of supernatants were scanned in the range of 350 nm to 750 nm in 1 nm steps, using a dual-beam spectrophotometer (Shimadzu UV/VIS UV1601 Spectrophotometer, Kyoto, Japan). The absorption spectra were analysed and pigment concentrations were quantified, employing the Gauss-Peak Spectra (GPS) method [79]. The sample spectrum was analysed, through the GPS fitting library, using SigmaPlot Software. This method is based on the sample spectrum fitting, by a linear combination, to the Gauss-peak spectra, which describes each pigment in the detected spectrum, identifying the samples pigment profile, chlorophyll *a*, chlorophyll *b*, auroxanthin, antheraxanthin, β-carotene, lutein, violaxanthin, and zeaxanthin. For a better evaluation of the light-harvesting and photoprotection mechanisms, the De-Epoxidation State (DES) was calculated as:

$$\mathrm{DES}=\frac{\left(\left[\mathrm{Antheraxanthin}\right]+\left[\mathrm{Zeaxanthin}\right]\right)}{\left(\left[\mathrm{Violaxanthin}\right]+\left[\mathrm{Antheraxanthin}\right]+\left[\mathrm{Zeaxanthin}\right]\right)}$$

### 4.8. Oxidative Stress Biomarkers

For enzyme extractions, leaf samples of *H. portulacoides* stored at −80 °C were used. Extractions were performed at 4 °C according to Tiryakioglu et al. [80] Frozen leaves were homogenized in 50 mM sodium phosphate buffer (pH 7.6) supplemented with 0.1 mM Na-EDTA in a ceramic mortar with a proportion of 500 mg (FW) to 8 mL respectively. The homogenate was centrifuged at 8890× *g*, 20 min at 4 °C, and the supernatant was transferred to a test tube and used for the antioxidant enzyme analyses.

The enzyme activity measurements of catalase (CAT, EC.1.11.1.6.), ascorbate peroxidase (APx, E.C. 1.11.1.11), guaiacol peroxidase (GPX, E.C. 1.11.1.7), and superoxide dismutase (SOD, E.C. 1.15.1.1) were performed in a dual-beam spectrophotometer (Shimadzu UV/VIS UV1601 Spectrophotometer) using quartz cuvettes. Catalase activity assays were performed according to the method of Teranishi et al. [81], by monitoring the H_2_O_2_ consumption and consequent decrease in absorbance at 240 nm (molar absorbance coefficient of 39.4 mM^−1^ cm^−1^). Ascorbate peroxidase was measured according to Tiryakioglu et al. [80], by observing the ascorbate oxidation and consequent absorbance reduction at 290 nm (molar extinction absorbance of 2.8 mM^−1^ cm^−1^). Guaiacol peroxidase measurement was performed according to Bergmeyer et al. [82], by monitoring guaiacol oxidation products formation and its increase in absorbance during 60 s at 470 nm (molar extinction absorbance of 26.6 mM^−1^ cm^−1^). Superoxide dismutase total activity was assayed according to the method of Marklund and Marklund [83], by measuring the oxidation rate of pyrogallol monitored at 325 nm. For reference, the autoxidation of pyrogallol was read without added enzyme extract in a parallel sample. Protein quantification was performed according to the Bradford method [84].

Quantification of peroxidation of membrane lipids was performed according to Heath and Packer [85]. First, leaf samples were homogenized in a freshly prepared Thiobarbituric acid (TBA) solution (0.5% (*w*/*v*) TBA in 20% (*w*/*v*) Trichloroacetic acid), in a proportion of 100 mg FW to 2 mL of solution. The homogenate was incubated for 30 min at 95 °C, cooled on ice to stop the reaction and centrifuged at 4000× *g*, 5 min at 4 °C. The absorbance was read at 532 nm and 600 nm in a Shimadzu UV-1601 spectrophotometer. Malondialdehyde (MDA) concentration was calculated using absorbance coefficient, 155 mM^−1^ cm^−1^ applying the following equation:

$$A_{532\;nm}-A_{600\;nm}=\left[MDA\right]mM\times \varepsilon MDA$$

### 4.9. Proline Quantification

Proline content was estimated according to [86]. The plant material was homogenized in 3% aqueous sulfosalicylic acid and the homogenate was centrifuged at 10,000 rpm, 15 min at 0 °C. The supernatant was used for proline determination. The reaction consisted of 2 mL of extract combined with 2 mL of glacial acetic acid and 2 mL of acidic ninhydrin solution. After incubation at 100 °C for 1 h, the reaction was stopped in an ice bath. The reaction mixture was extracted with 4 mL of toluene and its absorbance was read at 520 nm. The proline concentration, expressed in µmol g^−^^1^ FW, was estimated by comparison of measured data to a curve of different proline calibration standards.

### 4.10. Statistical Analysis

Boxplots with probability density of the data at different values smoothed by a kernel density estimator were computed and plotted using the ggplot2 package in R-Studio Version 1.4.1717. Non-parametric Kruskal–Wallis with Bonferroni post hoc tests, for comparisons between the variables and samples exposed to different exudate concentrations, were performed in R-Studio Version 1.4.1717 using the agricolae package [87].

## 5. Conclusions

Global soil salinization is an undeniable ongoing process, either due to exhaustive agricultural practices or increased marine flooding in coastal areas due to sea-level rise. Thus, it becomes of utmost importance to develop eco-friendly strategies to restore salinized soils. Halophytes are naturally adapted to salinized conditions; nevertheless, salt tolerance is not unlimited. Thus, the bioaugmentation of these species with marine PGPR can be seen as a new solution to this problem. The inoculation using a salt marsh PGPR consortium had a positive and significant impact on salt-stressed *H. portulacoides*. When comparing non-inoculated and inoculated plants, the most significant tolerance enhancements were found in mild or suboptimal salt stress conditions. This is mainly achieved through the improvement of the photochemical efficiency of the plants, with higher energy use efficiencies and lower energy dissipation. Even though, under severe salinity stress, the PGPR amelioration was less clear and observable effects were less significant, it had still a crucial impact on the maintenance of leaf water content, via the support of organic osmolytes production. This was expressed, for instance, by the significantly higher proline concentration. Additionally, a mitigation of the levels of oxidative stress parameters of the plants was also observed.

In summary, bioaugmented *H. portulacoides* could be seen as a candidate species to be used in the restoration of salinized soil, as well as a cash crop to be used in seawater agriculture. In both cases, the studied PGPR consortium will be a key component leading to a success of the respective project. Anyhow, more research is needed to explore in more detail the effectiveness, specificity, and limitations of these consortia of halophytes and the best matching plant-growth promoting rhizobacteria.

## Figures and Tables

**Table 1:** Summary of the bacteria isolates and their plant growth-promoting properties [24].

			Plant-Growth Promoting Traits					
Bacterial Strains	Sampling Site	Limit Salt Tolerance (mM)	P-Solubilization	Siderophore	ACC Deaminase (nm mg^−1^ h^−1^)	IAA (µg mL^−1^)	N-Fixation	EPS (OD_540_)
*Bacillus aryabhattai* SP20	Horta dos Peixinhos, Portugal	856	+	+	−	−	−	0.80 ± 0.01
*Stenotrophomonas rhizophila* EH7	Horta dos Peixinhos, Portugal	856	+	+	-	15.02 ± 0.31	+	−
*Pseudomonas oryzihabitans* RL18	Tagus Estuary, Portugal	1711	+	+	+* (26.9 ± 14.13)	39.55 ± 1.01	−	0.40 ± 0.01
*Salinicola endophyticus* EL13	Tagus Estuary, Portugal	1711	+	+	+*	71.35 ± 6.86	−	0.54 ± 0.01

− negative; +* visible growth on solid DF + ACC medium.

**Table 2:** Summary of fluorometric analysis parameters and their description.

Ψ0/(1 − Ψ0)	Contribution of the dark reactions from quinone A to plastoquinone
ΨEo/(1 − ΨEo)	The equilibrium constant for the redox reactions between PS II and PS I
RC/ABS	Reaction centre II density within the antenna chlorophyll bed of PS II
TR0/DI0	Contribution or partial performance due to the light reactions for primary photochemistry
SFI	Structure functional index for photosynthesis
SFI (NO)	Non-photosynthetic or dissipation structure functional index
ABS/CS	Absorbed energy flux per cross-section
TR/CS	Trapped energy flux per cross-section
ET/CS	Electron transport energy flux per cross-section
DI/CS	Dissipated energy flux per cross-section
RC/CS	The number of available reaction centres per cross-section

## Data Availability

Data available upon request to the authors.
